# Residential status and household wealth disparities in modern contraceptives use among women in Ghana: a cross-sectional analysis

**DOI:** 10.1186/s12905-023-02684-7

**Published:** 2023-10-24

**Authors:** Anthony Mwinilanaa Tampah-Naah, Elijah Yendaw, Joshua Sumankuuro

**Affiliations:** 1 Department of Geography, Simon Diedong Dombo University of Business and Integrated Development Studies, Wa, Post Office Box WA64 Ghana; 2 Department of Governance and Development Management, Faculty of Public Policy and Governance, Simon Diedong Dombo University of Business and Integrated Development Studies, Wa, Ghana; 3 Centre for Environment, Migration and International Relations, Simon Diedong Dombo, University of Business and Integrated Development Studies, Wa, Ghana; 4 Department of Public Policy and Management, Faculty of Public Policy and Governance, Simon Diedong Dombo University of Business and Integrated Development Studies, Wa, Ghana; 5 Centre for Health Policy, School of Public Health, Faculty of Health Sciences, University of the Witwatersrand, Johannesburg, South Africa; 6 School of Allied Health, Exercise and Sports Sciences, Faculty of Science and Health, Charles Sturt University, Orange, NSW Australia

**Keywords:** Residential status, Household wealth, Disparities, Contraceptive use, Trend, MICS, Ghana

## Abstract

**Background:**

Modern contraceptive refers to “a product or medical procedure that interferes with reproduction from acts of sexual intercourse”. The aim of this study was to assess the relationship between residential status and wealth quintile, and modern contraceptive use among women in Ghana.

**Methods:**

We examined residential status and wealth quintile on contraceptive use analysing the 2006, 2011 and 2018 Multiple Indicator Cluster Surveys datasets. A sample of 30,665 women in their reproductive ages (15–49 years) were enrolled in the surveys across Ghana. STATA version 13 was used to process and analyse the data. It examined socioeconomic and demographic characteristics, assessed modern contraceptive use prevalence among women, and used logistic regression models to determine predictors. The results were presented in odds ratio and adjusted odds ratio with 95% confidence intervals. All statistical tests were measured with p < 0.05.

**Results:**

In the three survey years, the highest prevalence of modern contraceptive usage was observed in 2011 (27.16%). The odds of using modern contraceptive increased by 19% in rural places (AOR = 1.19; 95% CI = 1.097–1.284) compared to urban places. The likelihood of women in second (AOR = 1.17; 95% CI = 1.065–1.289), middle (AOR = 1.24; 95% CI = 1.118–1.385), and fourth (AOR = 1.25; 95% CI = 1.113–1.403) wealth quintile using contraceptives increased compared to those of low wealth quintile. With the interactive terms, rural-second (AOR = 1.38; 95% CI = 1.042–1.830), rural-middle (AOR = 1.45; 95% CI = 1.084–1.933), rural-fourth (AOR = 1.52; 95% CI = 1.128–2.059), and rural-high (AOR = 1.42; 95% CI = 1.019–1.973) were more likely to use contraceptives compared to urban-low women. Despite lower odds, women of the age groups 20–24 (AOR = 2.33; 95% CI = 2.054–2.637), cohabitaing (AOR = 1.07; 95% CI = 0.981–1.173), secondary or higher education (AOR = 1.55; 95% CI = 1.385–1.736), Central (AOR = 1.48; 95% CI = 1.296–1.682) and Eastern (AOR = 1.48; 95% CI = 1.289–1.695) regions significantly predicted modern contraceptive use.

**Conclusion:**

Modern contraceptive use in Ghana is low. Women in rural-rich categories are more likely to use modern contraceptives. Background factors such as age, marital status, educational attainment, and previous child experiences predict modern contraceptive use. It is recommended for the intensification of contraceptive awareness and utilization for all reproductive age women, regardless of education, marriage, or wealth.

## Background

Global campaigns to improve maternal health outcomes have led to the proliferation of different health interventions. For example, when maternal mortality was declared a national emergency across Ghana, the government recognised family planning as a multisectoral issue with benefits beyond health [1, 2]. Contraceptives are used in family planning as a conscious effort by a couple to limit or space the number of children they want [3, 4]. Hubacher and Trussell have categorised contraceptives into ‘modern’ and ‘traditional [5]. Traditional and modern contraception methods are currently known and aim to improve women’s health, financially empower households and women [6].

In low-and middle-income countries (LMICs), modern contraceptives significantly lower fertility rates [7, 8]. Additionally, scientific research has shown that modern contraceptive techniques are more successful than traditional ones at preventing unintended births [9, 10]. This makes modern contraceptive use a critical public health intervention and a cost-effective strategy to reduce maternal mortality, avert unintended pregnancies, and control unprecedented population growth, especially in low-middle-income countries, including Ghana [7, 8].

Programmatic efforts continued in Ghana until the National Population Council was formed in 1992 and subsequent scale-up of interventions after the 1994 International Conference on Population and Development in Cairo [2, 11]. Contraceptive use was legitimised and added to the National Health Insurance Basic Benefits Package through a legislative instrument in 2017 to enhance access to services and commodities under the national health insurance scheme [12, 13]. Ghana’s government’s efforts were galvanised by preparing and launching the Family Planning Costed Implementation Plan (GFPCIP, 2016–2020), which was launched in September 2015 [12, 13]. The plan primarily aimed to increase the number of women using modern contraception from approximately 1.5 million in 2015 to 1.9 million by December 2020. Moreover, the contraceptive prevalence rate among married women was projected to increase from 22.2% to 2015 to 29.7% in 2020; and from 31.7 to 40% among unmarried women within same period [12].

Despite these commitments, the country still recorded relatively low modern contraceptives uptake among Ghanaian women ranging from 15.7% to 2008 to only 21.5% in 2014. The Multiple Indicator Cluster Survey (MICS, 2017–2018) recently observed starkly low utilisation of the various contraceptive methods [14]. Nationwide, injectables recorded the highest of 38.4%, pills (24.3%), implant (21.6%), IUD (4.3%), female sterilisation (7%), male condom use (3.8%), while other miscellaneous methods accounted for only 0.5% of the population [14]. Different factors may explain the low uptake of modern contraceptives. From the Demographic and Health Survey Data for Nigeria, Lamidi [15] reported that higher educational attainment, autonomy in healthcare decision-making, and earning good income were significantly associated with contraceptive use. Thus, educated women were four times more likely to use modern contraceptives [3, 4]. Similarly, educated women demonstrated increased acceptance and utilisation of modern contraceptives compared to uneducated women in Bangladesh [16].

Indeed, unavoidable barriers related to geography influenced women’s access to modern contraception. Rural women do not have full authority to utilise contraceptives on time, and some of these are related to cultural beliefs and misperceptions. For example, in the Upper West region, men force women to the health facility to remove IUDs [17]. Overall findings on contraceptive use in Ghana and other low-middle-income settings are hinged on various factors of socioeconomic, demographic, and contextual variables (for example, distance and location of health facilities). Amissah and colleagues [18] reported high cost of modern contraceptives in Ghana subjecting women, especially, rural-poor women multiple burden access them [10, 18]. Indeed, low access and poor contraceptive use has mortality and clinical implications including increased likelihood of using unapproved contraceptives, unsafe abortions arising from unplanned and unwanted pregnancies [19]. Given the disparities in factors associated with modern contraceptive use in Ghana; and the fact that most communities are rural, this may present a significant challenge to stakeholder efforts towards increasing contraceptive use, prevalence, and the overall attainment of Sustainable Development Goal (SDG) 3 [3].

Further, a thorough search of the extant literature indicates that there is no known recent national-level study into populations’ residential status and wealth quintile on contraceptive use in Ghana. We thus aimed to comprehensively examine the relationship between residential status and wealth quintile, and modern contraceptive from analysis of the 2006, 2011 and 2018 MICS datasets. The findings of this study are important as they have the potential to guide key national policy decisions in healthcare delivery including the design and implementation of maternal health and family planning programs in Ghana and similar LMICs.

## Materials and methods

### Data source and study group

The study used secondary data from three rounds of Multiple Indicator Cluster Survey (MICS) of the years 2006, 2011, and 2018 rolled out in Ghana [20–22]. These are cross-sectional, nationally representative surveys. MICS specifically measure health indicators that allow countries to collect data for use in policies, programmes, and national development plans. The survey sample was selected using a multi-stage, stratified cluster approach. More details on the survey are published elsewhere [23]. A pooled sample of 30,665 women in their reproductive ages (15–49) were included in this study (Table 1). Specifically, 6240, 10,963, and 13,462 respectively were sub-sampled from each survey year (2006, 2011, and 2018).

### Dependent variable

The dependent variable used in this study was on whether women were currently using any modern contraceptive. This was captured in the survey as ‘Currently using a method to avoid pregnancy’. This variable has two responses and they were dichotomised as ‘yes = 1’for women who used modern contraceptive and ‘no = 0’ for those who did not use any modern contraceptive. Modern contraceptive methods were categorised according to Hubacher and Trussel’s proposed definition: “a product or medical procedure that interferes with reproduction from acts of sexual intercourse” [5]. The modern methods embedded in the definition and found in the MICS are sterilisation (male or female), intrauterine device (IUD), injectable, implant, pills, condom (male or female), diaphragm and foam/jelly. These modern contraceptive definitions and categories have been applied in similar studies [24, 25].

### Independent variables

The main independent variable was place of residence and wealth index quintile. These variables were interactively treated consisting of urban wealth index quintile and rural wealth index quintile. In terms of quintiles, responses to either of these variables were ‘low, second, middle, fourth, and high’. Other potential confounding demographic and socioeconomic independent variables were simultaneously controlled. The variables and how they were measured are place of residence (urban, rural) [4], wealth index quintile (low, second, middle, fourth, high) [2], age (15–19, 20–24, 25–29, 30–34, 35–39, 40–44, 45–49) [11], marital status (currently married, living with a man, no union) [9], educational level (none, primary, middle/junior secondary school [JSS]) [4], region (Western, Central, Greater Accra, Volta, Eastern, Ashanti, Brong Ahafo, Northern, Upper East, Upper West) [5], and ever given birth (yes, no) [12]. Further, selection of these variables were based on theoretical and practical relevance in related literature.

### Data analyses

The data was analysed in three stages using frequencies, percentages, chi-squre, and logistic regression tests. First, socioeconomic and demographic characteristics (age, marital status, educational level, region, and ever given birth) were cross-tabulated with modern contraceptive use to examine statistically significant factors. Second, the prevalence of modern contraceptive use was assessed among the women. Third, the main analysis, logistic regression models were employed to determine predictors of modern contraceptive use. Two models (Model I, and Model II) were generated using all independent variables considered for this study. In Model I, bivariate analysis was done to ascertain associations between the various dependent and independent variables. Added to this model were interactions regarding place of residence (urban, rural), and wealth quintile (low, second, middle, fourth, high). With Model II, a full modelling technique was used to appraise the combined effects on each variable on modern contraceptive use among women. All statistical tests were measured with *p* < 0.05. Logistic regression results were presented in odds ratio (OR) and adjusted odds ratio (AOR) with their corresponding 95% confidence intervals (CI). Due to the complex sampling design of the MICS in relation to clustering and stratification of samples, the appropriate sample weight (*wmweight*) for women was applied to reconfigure the analysis to ensure the results reflect the respondents for purposed of effective generalisation. The Stata command used to set sample weights was ‘*svyset [pw = wmweight]’*. Then all bivariate and multivariate analysis commands in Stata were prefixed with the sample weight command (*svy*:). Data processing and mining were accomplished using STATA version 13. This study used the STROBE cross-sectional reporting guidelines [26].

## Results

### Prevalence and correlates of modern contraceptive use

The independent variables used to examine their association with modern contraceptive use were statistically significant (Table 1). These variables are place of residence, wealth index quintile, age, marital status, educational level, region, and ever-given birth. In all the survey years, a higher proportion of women in rural places (23%) reported of using a modern contraceptive. Minimal proportional differences were observed for the wealth index quintile. However, slightly higher usage of contraceptives was linked with those of middle (24%) and fourth (24%) wealth index quintile. With age, women in their mid-reproductive ages (30–34 years) mostly used contraceptives (30%). Equally, those currently living with men (34%) mostly use contraceptives. Also, women who attained primary (23%), and middle/junior secondary school (23%) education mostly used contraceptives. Regionally, modern contraceptive usage was higher in Central (29%), and Eastern regions (28%). Women who had ever given birth (27%) had a higher proportion of contraceptive use.

**Table 1:** Socioeconomic and demographic characteristics by modern contraceptive use

Characteristic	Modern contraceptive use			
	2006	2011	2018	All surveys
Place of residence	p < 0.001*	p < 0.001*	p < 0.001*	p = 0.025*
Urban	356(15.73)	1141(30.11)	1222(18.40)	2719(21.42)
Rural	400(12.35)	1494(25.16)	1703(24.97)	3597(22.52)
Wealth index quintile	p < 0.001*	p < 0.001*	p < 0.001*	p < 0.001*
Low	99(7.91)	647(18.84)	708(22.64)	1454(18.61)
Second	144(12.89)	512(27.93)	552(24.61)	1208(23.26)
Middle	127(13.64)	513(33.71)	566(22.48)	1206(24.26)
Fourth	182(17.72)	479(32.00)	580(22.48)	1241(24.41)
High	204(17.36)	484(34.20)	519(17.22)	1207(21.54)
Age	p < 0.001*	p < 0.001*	p < 0.001*	p < 0.001*
15–19	79(6.76)	212(10.81)	202(6.99)	493(8.19)
20–24	150(16.50)	436(32.51)	634(24.04)	1220(24.96)
25–29	158(18.39)	548(38.51)	523(28.21)	1229(29.71)
30–34	134(17.77)	491(35.12)	513(32.47)	1138(30.49)
35–39	127(17.89)	427(31.94)	477(29.70)	1031(28.22)
40–44	79(14.18)	316(27.41)	352(23.16)	747(23.13)
45–49	29(5.33)	205(18.86)	224(16.30)	458(15.24)
Marital status	p < 0.001*	p < 0.001*	p < 0.001*	p < 0.001*
Currently married	433(15.67)	1500(31.70)	1603(28.47)	3536(26.97)
Living with a man	110(21.96)	505(37.30)	514(34.82)	1129(33.89)
Not union	213(9.52)	630(17.36)	808(12.71)	1651(13.51)
Educational level	p < 0.001*	p < 0.001*	p < 0.001*	p < 0.001*
None	179(9.68)	790(22.69)	704(24.52)	1673(20.39)
Primary	134(12.93)	504(28.12)	528(24.08)	1166(23.38)
Middle/JSS	317(17.39)	880(28.12)	1098(21.96)	2295(23.06)
Secondary or more	126(15.97)	461(34.64)	595(17.51)	1182(21.42)
Region	p < 0.001*	p < 0.001*	p < 0.001*	p < 0.001*
Western	45(8.96)	199(33.67)	280(22.56)	524(22.45)
Central	72(17.91)	534(36.88)	275(22.45)	881(28.65)
Greater Accra	160(19.46)	298(37.77)	324(19.01)	782(23.59)
Volta	34(9.91)	144(21.51)	255(21.16)	403(19.39)
Eastern	93(17.51)	204(36.56)	394(29.49)	691(28.49)
Ashanti	112(14.64)	201(27.16)	388(20.61)	701(20.69)
Brong Ahafo	52(12.44)	218(36.15)	300(24.83)	570(25.57)
Northern	72(10.18)	326(19.11)	170(12.58)	568(15.09)
Upper East	80(14.13)	228(17.63)	261(24.35)	569(19.46)
Upper West	36(8.09)	313(21.69)	278(22.35)	627(20.02)
Ever given birth	p < 0.001*	p < 0.001*	p < 0.001*	p < 0.001*
Yes	589(15.77)	2189(31.63)	2526(28.31)	5304(27.09)
No	167(9.45)	446(16.04)	399(8.79)	1012(11.14)
*N*	6240	10,963	13,462	30,665

*significant (p < 0.05); figures outside the parenthesis are the number of women who use modern contraceptives and figures in the parenthesis are their proportions

**Fig. 1:**
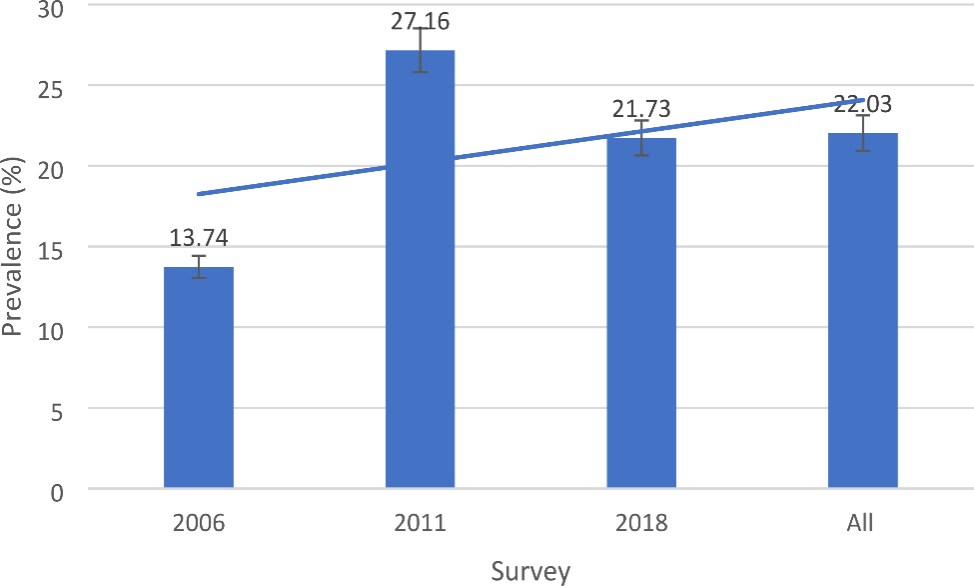
Prevalence of modern contraceptive usage

A prevalence rate of 22.03% of modern contraceptive use was recorded for all survey years (Fig. 1). In terms of percentages, the usage of contraceptives was highest in 2011 (27.16%).

Table 2 shows modern contraceptive usage and types across the survey years. The modern contraceptives used in this study are female sterilisation, male sterilisation, pill, IUD, injectable, implant, male condom, female condom, diaphragm, and foam/jelly. Comparing the two forms of sterilisation (for females and males), more females (0.07%) opted for their form than males (0.01%). Again, across all the survey years, injectable (7.15%) was mostly used and pill (5%) was the next preferred modern contraceptive. Also, usage of implant (2.94%) and male condom (2.44%) were equally common among the women. Relatively, the least modern contraceptives used by the women were IUD (0.49%), female condom (0.15%), diaphragm (0.05%), and form/jelly (0.07%).

**Table 2:** Modern contraceptive use and types across years of survey

Contraceptive usage	2006		2011		2018		All surveys	
	Yes	No	Yes	No	Yes	No	Yes	No
Currently using a method to avoid pregnancy	756 (13.74)	4,745 (86.26)	2635 (27.16)	7066 (72.84)	2925 (21.73)	10,537 (78.27)	6316 (22.0 3)	22,348 (77.97)
Modern contraceptives								
Female sterilisation	14 (0.25)	5,487 (99.75)	65 (0.67)	9636 (99.33)	122 (0.91)	13,340 (99.09)	201 (0.70)	28,463 (99.30)
Male sterilisation	0 (0.00)	5,501 (100.00)	2(0.02)	9699 (99.98)	2 (0.01)	13,460 (99.99)	4 (0.01)	28,660 (99.99)
Pill	197 (3.58)	5,304 (96.42)	554 (5.71)	9147 (94.29)	565 (4.20)	12,897 (95.80)	1316 (4.59)	27,348 (95.41)
IUD	15 (0.27)	5,486 (99.73)	31 (0.32)	9670 (99.68)	95 (0.71)	13,367 (99.29)	141 (0.49)	28,523 (99.51)
Injectable	242 (4.40)	5,259 (95.60)	741 (7.64)	8960 (92.36)	1066 (7.92)	12,396 (92.08)	2049 (7.15)	26,615 (92.85)
Implant	21 (0.38)	5,480 (99.62)	139 (1.43)	9562 (98.57)	683 (5.07)	12,779 (94.93)	843 (2.94)	27,821 (97.06)
Male condom	164 (2.98)	5,337 (97.02)	426 (4.39)	9275 (95.61)	109 (0.81)	13,353 (0.81)	699 (2.44)	27,965 (97.56)
Female condom	22 (0.40)	5,479 (99.60)	12 (0.12)	9689 (99.88)	9 (0.07)	13,453 (99.93)	43 (0.15)	28,621 (99.85)
Diaphragm	1 (0.02)	5,500 (99.98)	4 (0.04)	9697 (99.96)	8 (0.06)	13,454 (99.94)	13 (0.05)	28,651 (99.95)
Form/Jelly	8 (0.15)	5,493 (99.95)	10 (0.10)	9691 (99.90)	1 (0.01)	13,461 (99.99)	19 (0.07)	28,645 (99.93)

### Predictors of modern contraceptive use

Table 3 depicts logistic regression analysis of the predictors of modern contraceptive use among women in Ghana. Two models (Model I and Model II) were generated to ascertain their likelihood of using modern contraceptives. Model I contained bivariate logistic outcomes while potential confounding variables were controlled for in Model II. In both models, all the variables had statistically significant categories associated with contraceptive use. They are place of residence, wealth quintile, age, marital status, education level, region, and ever-given birth. In addition to these, interaction terms (urban/rural) equally proved to be linked with the outcome variable (contraceptive use).

**Table 3:** Logistic Regression analyses determining modern contraceptive use (All surveys)

Characteristic	Model I		Model II	
	OR (95% CI)	*p*-value	AOR (95% CI)	*p*-value
Place of residence Urban Rural	1 1.07(1.008-1.128)	0.025	1 1.19(1.097-1.284)	< 0.001
Wealth index quintile Low Second Middle Fourth High	1 1.33(1.216-1.444) 1.40(1.284-1.526) 1.41(1.296-1.538) 1.20(1.102-1.307)	0.000 0.000 0.000 0.000	1 1.17(1.065-1.289) 1.24(1.118-1.385) 1.25(1.113-1.403) 1.06(0.928-1.208)	0.001 < 0.001 < 0.001 0.392
Interaction terms Urban#low Urban#second Urban#middle Urban#fourth Urban#high Rural#low Rural#second Rural#middle Rural#fourth Rural#high	1 1.30(0.962-1.758) 1.44(1.091-1.902) 1.53(1.161-2.004) 1.35(1.032-1.773) 1.19(0.908-1.553) 1.63(1.244-2.139) 1.88(1.427-2.482) 2.07(1.553-2.756) 1.90(1.396-2.612)	0.087 0.010 0.002 0.029 0.208 0.000 0.000 0.000 0.000	1 1.25(0.911-1.702) 1.28(0.961-1.716) 1.24(0.933-1.652) 1.04(0.780-1.389) 1.19(0.904-1.575) 1.38(1.042-1.830) 1.45(1.084-1.933) 1.52(1.128-2.059) 1.42(1.019-1.973)	0.169 0.090 0.137 0.773 0.212 0.025 0.012 0.006 0.038
Age 15-19 20-24 25-29 30-34 35-39 40-44 45-49	1 3.73(3.333-4.175) 4.74(4.230-5.311) 4.92(4.382-5.521) 4.40(3.922-3.815) 3.37(2.982-3.915) 2.02(1.760-2.309)	0.000 0.000 0.000 0.000 0.000 0.000	1 2.33(2.054-2.637) 2.32(2.026-2.667) 2.19(1.892-2.528) 1.95(1.687-2.264) 1.48(1.269-1.724) 0.90(0.764-1.059)	< 0.001 < 0.001 < 0.001 < 0.001 < 0.001 0.207
Marital status Currently married Cohabiting Not union	1 1.39(1.279-1.506) 0.42(0.396-0.451)	0.000 0.000	1 1.07(0.981-1.173) 0.56(0.513-0.614)	0.123 < 0.001
Educational level None Primary Middle/JSS Secondary +	1 1.19(1.094-1.296) 1.17(1.089-1.256) 1.06(0.978-1.157)	0.000 0.000 0.147	1 1.26(1.142-1.380) 1.42(1.301-1.556) 1.55(1.385-1.736)	< 0.001 < 0.001 < 0.001
Region Western Central Greater Accra Volta Eastern Ashanti Brong Ahafo Northern Upper East Upper West	1 1.39(1.224-1.571) 1.07(0.940-1.209) 0.83(0.718-0.962) 1.38(1.207-1.569) 0.90(0.792-1.024) 1.19(1.035-1.359) 0.61(0.537-0.700) 0.83(0.730-0.953) 0.86(0.759-0.985)	0.000 0.317 0.013 0.000 0.111 0.014 0.000 0.008 0.029	1.48(1.296-1.682) 1.29(1.129-1.485) 0.88(0.714-0.967) 1.48(1.289-1.695) 0.97(0.851-1.111) 1.33(1.153-1.531) 0.66(0.572-0.763) 1.01(0.869-1.167) 0.98(0.855-1.139)	< 0.001 < 0.001 0.017 < 0.001 0.683 < 0.001 < 0.001 0.923 0.864
Ever given birth Yes No	1 0.34(0.313-0.362)	0.000	1 0.52(0.469-0.584)	1 < 0.001
Number of observations			28,660	
Prob > chi2			0.0000	
Pseudo R2			0.0771	
Hosmo-Lemeshow			Chi2(8) = 15.92	
Mean VIF			2.80	

AOR: Adjusted odds ratio; *significant (p < 0.05); CI: Confidence interval

The multivariate logistic regression analysis (Model II) revealed statistically significant associations of place of residence, wealth index quintile, and their interactive terms with modern contraceptive use (Table 3). All the other confounding variables (age, marital status, educational level, region, and ever given birth) were included in the multivariate model (Model II) to assess their possible combined effects on the outcome variable (contraceptive use). The odds of using modern contraceptive increased in rural places (AOR = 1.19; 95% CI = 1.097–1.284) compared to urban places. The likelihood of women in second (AOR = 1.17; 95% CI = 1.065–1.289), middle (AOR = 1.24; 95% CI = 1.118–1.385), and fourth (AOR = 1.25; 95% CI = 1.113–1.403) wealth quintile using contraceptives increased compared to those of low wealth quintile. With the interactive terms, rural-second (AOR = 1.38; 95% CI = 1.042–1.830), rural-middle (AOR = 1.45; 95% CI = 1.084–1.933), rural-fourth (AOR = 1.52; 95% CI = 1.128–2.059), and rural-high (AOR = 1.42; 95% CI = 1.019–1.973) were more likely to use contraceptives compared to urban-low women. Despite lower odds, women of the age groups 20–24 (AOR = 2.33; 95% CI = 2.054–2.637), 25–29 (AOR = 2.32; 95% CI = 2.026–2.667), and 30–34 (AOR = 2.19; 95% CI = 1.892–2.528) were more than two times likely to use modern contraceptives compared to women aged 15–19 years. Women not in union (AOR = 0.56; 95% CI = 0.513–0.614) had lower likelihood of using contraceptives compared to married couples. The possibility of using contraceptives increased with women who had primary (AOR = 1.26; 95% CI = 1.142–1.380), middle or junior secondary school (AOR = 1.42; 95% CI = 1.301–1.556), and secondary or higher education (AOR = 1.55; 95% CI = 1.385–1.736) compared to those with no education. Likewise, increased odds for the usage of contraceptives were recorded for women in Central (AOR = 1.48; 95% CI = 1.296–1.682), Greater Accra (AOR = 1.29; 95% CI = 1.129–1.485), Eastern (AOR = 1.48; 95% CI = 1.289–1.695), and Brong Ahafo (AOR = 1.33; 95% CI = 1.153–1.531) regions. Women in Volta (AOR = 0.88; 95% CI = 0.714–0.967), and Northern (AOR = 0.66; 95% CI = 0.572–0.763) regions were less likely to use contraceptives. Still, women who had never given birth (AOR = 0.52; 95% CI = 0.469–0.584) were less likely to use a modern contraceptive compared to those who ever gave birth (Table 3).

## Discussion

This study aimed to comprehensively examine the relationship between residential status and wealth quintile, and modern contraceptive use, from analysis of the 2006, 2011 and 2018 MICS datasets for Ghana. Thus, we sought to determine women’s place of residence and wealth quintile on contraceptive use in Ghana. The modern contraceptives used in this study are female sterilisation, male sterilisation, pill, IUD, injectable, implant, male condom, female condom, diaphragm, and foam/jelly. In terms of percentages, the usage of contraceptives was highest in 2011 (27.16%) and only 22.03% for 2006, 2011 and 2018 combined. Overall, the analysis found that age, marital status, wealth quintile, educational level, region of residence, ever given birth significantly predicted modern contraceptive use in this study.

Over the years, studies have found a strong association between wealth quintiles and modern contraceptive use. However, very few studies have considered women’s residential status, wealth quintile and contraceptive use in Ghana [4, 12, 27]. Thus, we have presented the discussion of the findings in two strands. First, we considered the geographic location and wealth quintile of participants and finally, the socio-demographic and obstetric characteristics of participants versus modern contraceptive use were discussed.

### Place of residence, wealth quintile and modern contraceptive use

From the results, the prevalence of contraceptive use was estimated at 23% (rural) and 21.42% (urban) [28], which is similar to the 22% reported by the Ghana Demographic and Health Survey (GDHS), but slightly below the 23.3% target for the Ghana Health Service [29] contraceptive use. Although this is higher than Nigeria (12.8–18.0%) [30, 31] and Togo (21%) [6], it is far less than countries with much slower economic growth like Malawi (58% in 2016) [32] and Ethiopia (41.4% in 2019) [12, 15]. While the difference of use of modern contraceptive among rural and urban women was not much in this study, it must be noted that only 21.42% of the women had secondary or higher educational attainment – a level, that will grant them access to relevant information and knowledge about contraceptive methods. Similarly, women in rural areas may have lower levels of education and awareness about family planning methods, leading to a higher reliance on modern contraceptives for birth control. However, women in urban areas may have more access to information and resources, allowing them to explore a wider range of family planning options [Table 1]. The Community-based Health Planning Services (CHPS) compounds concept – also known as clinics, in Ghana, conducts door-to-door campaigns about maternal and child health in the rural. This may have had a greater impact on rural areas, where access to healthcare services and information was previously limited [4, 6]. In addition, the literature has shown that geographical barriers to access modern contraception do exist. For example, women who resided in some regions in Ghana such as Central, Greater Accra, Eastern, and Brong Ahafo showed higher likelihood of modern contraceptives use when compared with women from Volta and Northern Regions. It is worth noting that traditional beliefs and practices prevalent in the northern and Volta regions may help explain the non-acceptability of modern contraceptive methods. Although the literature identifies distances to health facilities and other modern contraceptives services providers, it is sad to understand that accessing skilled maternal healthcare was problematic in some communities in these regions, due to unfavourable cultural beliefs and limited women’s autonomy [33]. These regions are dominated by patriarchal households, with women having little say in decisions pertaining to the number of children couples would want to produce, birth spacing, and modern contraceptives use [33–35]. Therefore, women with little autonomy may not take up any modern contraceptive methods for fear of spousal backlash [36]. It is particularly an important determinant since most of the populations in low-middle-income settings reside in rural places [12, 35, 37].

Furthermore, the data on the wealth quintile allows us to examine equity issues, and we found significant disparities in modern contraceptive use among the rich and poor. The results reveal that 24% of women within the middle wealth class and fourth wealth index reported the highest proportions among women who utilised contraceptives. However, significant disparities exist across the quintiles and periods. This prevalence rate corroborates with findings from earlier studies in Ghana and elsewhere [4, 38, 39]. Hounton and colleagues observed similar trends among adolescents aged 15–19 years in Burkina Faso, Nigeria, and Ethiopia [38]. Accordingly, those within the highest wealth quintiles significantly used more modern contraception than their peers in the lowest wealth quintiles. Thus, women’s wealth position is a significant predictor of modern contraceptive use in this study. Specifically, women of the middle and fourth wealth quintiles showed 59–60% odds of modern contraceptives use compared with those of the second quintiles [see Table 3]. However, it was intriguing to observe that rural-fourth women (OR = 2.07; 95% CI = 1.553–2.756) were two times more likely to use a modern contraceptive than urban-low women [see Table 3]. The literature has further shown that most rural residents may have reduced knowledge, creating misperceptions about contraceptive methods and their effects. These have significantly impacted acceptability and prevalence rates [4, 15, 39]. Findings from a comparative analysis of 2003, 2008 and 2014 Demographic and Health Survey data showed that women’s place of residence and educational attainment significantly predicted modern contraceptives use in Ghana [4].

The results confirm earlier findings in other countries [39, 40]. For instance, an analysis of the 2010 DHS dataset for Malawi reported that 82.4% of women within the high wealth quintile demonstrated modern contraceptives use compared to 66.8% of women in the low wealth cohort [39]. The finding also supports previous results in a large study involving 23 countries in Latin America and the Caribbean [40]. Low use of modern contraceptives among women of low wealth quintiles was found in the same study. Overall, the findings reveal that quite a good proportion of women across the different wealth quintiles have shown the prevalence of modern contraceptive use. While there exist differential proportions for the various survey periods, the findings showed significant improvements of women within the low wealth brackets when compared to those in the other cohorts. Specifically, the proportions for modern contraceptives use among the low wealth quintile were 7.91% (2006), 18.84% (2011), which further increased to 22.64% in 2018 [see Tables 1 and 3]. It is quite commendable that the improvement and increased utilisation of contraceptives in rural places may be due to intensified maternal education through door-to-door visitation, media education, maternal education and possibly due to the ongoing proliferation of the community health-based planning services (CHPS) compounds initiatives in rural communities in Ghana [41]. These findings contrast with earlier studies in Nigeria [39]. The authors revealed that rural women had low knowledge of modern contraception, which could affect decision-making and, thus, their acceptability. Arguably, increased family planning counselling at the antenatal clinics may have increased women’s intentions to use contraceptives. Indeed, recent evidence also suggests women in rural places may have inadequate autonomy to decide on contraceptive use, contrary to the present findings [33]. While disparities in the findings may result from several contextual, social/cultural, and economic factors, it does suggest policy specific strategies implemented by the Ghana Health Service have had significant effects, over time.

### Socio-demographic and obstetric related factors and modern contraceptive use

The findings showed that age was a significant predictor of contraceptive use. Across all survey years, the results indicate that the current use of modern contraceptives existed among women of the various age cohorts. For example, women aged 20–24, 25–29, 30–34, and 40–44 years were more likely to use modern contraceptives than younger women (15–19) and the much older ones of 45–49 years. However, women aged 30–34 were 4.9 times (OR = 4.92; 95% CI = 4.382–5.521) more likely to use modern contraceptives when compared with the other age cohorts [see Table 3]. These findings resonate with earlier findings [38, 39]. The finding suggests that women within these age cohorts may have experienced childbirth and potentially received antenatal education. Meanwhile, younger women may not be married as yet and may not have the chance to participate in any educational session on contraceptive use, which could suggest their low utilisation of modern contraception. It is possible they may as well be deterred by socio-cultural values, attitudes of health personnel and cost given their age as dependents [42].

A multi-country study involving Ethiopia, Nigeria and Burkina Faso similarly revealed low contraceptive use among sexually active women of the ages 15–19 [38]. However, 8.19% prevalence among women within this age bracket is far from expectation when compared with the prevalence rate for same ages in countries such as Burkina Faso (11.2%), and Ethiopia (24.1%) [39] It is worth noting that modern contraceptives use may involve some financial obligations on the users, particularly when such services are not free. Younger women of 15–19 years in Ghana may not be gainfully employed to cater for their reproductive healthcare needs, including purchasing contraceptives. Thus, this could help explain the low uptake among such ages. The literature reveals that free contraceptives predisposed women to use them [24, 35]. Although the cost of modern contraceptives has been significantly reduced to increase financial access, such products may be found in public health facilities, which may not be geographically accessible to all women.

Besides the age, the results have demonstrated that women who were not in marital union were less likely to utilise modern contraceptives. Specifically, there was a reduction in prevalence from 17.36% to 2011 to 12.71% in 2018 [see Table 1]. The finding corroborates with previous findings in Nigeria, Ghana, and Ethiopia [4, 37, 38].

In terms of educational attainment, women with some formal schooling and basic education showed significant interest in using modern contraceptive. However, there was remarkable uptake among women with secondary education and higher (55%) compared to those with primary (26%), middle or junior secondary school (42%). The increase odds of modern contraceptives use among well-educated women compared to women with no educational backgrounds is reflected in the sharp decrease in education-related inequality in modern contraceptive use between 2006 and 2018. This finding confirms a multilevel analysis of 1988 to 2008 GDHS dataset [27]. The study revealed significant disparities in modern contraceptives use among women with low education and those with some level of education. However, the positive effect of educational attainment is well documented in previous studies in Ghana and similar settings [12, 13, 15, 27, 35, 37]. Thus, women of different educational levels may use modern contraceptives for different reasons, be it for limiting or spacing childbirth, different fertility preferences irrespective of their access to modern contraceptives or it may be due to lack of access to information and services to prevent high-risk births which carries along with the health and moral implications in the case of unwanted fertility. Besides these, higher educational attainment may provide women with accurate knowledge about contraception, contraceptive options and understand the benefits that can be derived from it. Also, the long duration required by higher educational attainment may in itself encourage sexually active young women to use modern contraception as they seek to postpone pregnancy until after school or perhaps employment.

Finally, ever given birth was also significantly associated with modern contraceptives utilisation in this study. The finding is consistent with previous studies in Ghana, Nigeria and Bangladesh [12, 16, 27, 39]. Specifically, in Bangladesh, modern contraceptive use was lowest among women who had no children. While we recommend qualitative studies into the reasons, it is understandable that this class of women will need to give birth before considering birth control or birth space measures. Evidently, women who have never given birth may have a mixture of biological and social misconceptions of the potential effect of modern contraceptives on their future childbearing decisions [43].

### Strengths and limitations

This study has some methodological strengths. The study used three different sets of nationally representative survey data to examine how women of various reproductive age categories use modern contraceptives. Key importance of this study lies in its usage of two important factors (residence and wealth quintile) interactively to measure their influences on the usage of modern contraceptives across Ghana. Also, the usage of nationally representative survey data implies that the findings of this study reflect what pertains in the Ghanaian population. It must be noted that the study has some limitations. The cross-sectional nature of the MICS data limits its usage to determine causalities between variables. Also, there may be the issue of over- or under-estimating the use of modern contraceptives during the last sexual encounter since the related question might had been sensitive for disclosure. In addition, Interpretation of results would have been adequate if some qualitative data were collected from sections of women to triangulate with the statistical findings. However, authors have extant knowledge and experience from the literature and extensive research in women’s health and related subjects and have embedded these in the analysis.

## Conclusion

Modern contraceptive use in Ghana is low, with rural areas having a slightly higher concentration. Women in rural-rich categories are more likely to use modern contraceptives compared to those in lower wealth quintiles residing in urban suburbs. Increasing access to modern contraception for rural and economically vulnerable populations could improve access and use. It is recommended for Ghana Health Service and related health organizations in the country to intensify contraceptive awareness and utilization for all reproductive age women, regardless of the place of residence, education, marriage, or wealth. Qualitative research is further recommended to explore women’s experiences accessing and using modern contraceptives in rural areas and the relative low usage among urban women in Ghana.

## Data Availability

The data that support the findings of this study are available from UNICEF repository upon request by individual researchers for specific purposes. UNICEF reviews each request before approval and access is granted to download and use the data. Hence, researchers interested in the data used for this study can equally contact UNICEF (https://mics.unicef.org/surveys) for permission to use the data.

## List of abbreviations

UNICEF: United Nations Children Education Fund
MICS: Multiple Indicator Cluster Survey
IUD: Intrauterine device
GFPCIP: Ghana Family Planning Costed Implementation Plan
LMIC: Low-middle-income countries
SDG: Sustainable Development Goals
OR: Odd ratio
AOR: Adjusted odd ratio
CI: Confidence interval
GDHS: Ghana Demographic and Health Survey
DHS: Demographic and Health Survey
CHPS: Community health-based planning services
